# Effect of oral gargle containing *Lespedeza cuneata* extract on periodontal health improvement and disease prevention: a randomized, controlled clinical trial

**DOI:** 10.1186/s12903-023-02816-3

**Published:** 2023-02-21

**Authors:** Yu-Rin Kim, Seoul-Hee Nam

**Affiliations:** 1 Department of Dental Hygiene, Silla University, 140 Baegyang-daero 700beon-gil, Sasang-gu, Busan, 46958, Republic of Korea; 2 Department of Dental Hygiene, College of Health Sciences, Kangwon National University, 346 Hwangjo-Gil, Dogye-Up, Samcheok-Si, Gangwon-Do 25945 Republic of Korea

**Keywords:** Periodontal health, *Lespedeza cuneata*, Natural extract, Clinical study

## Abstract

**Background:**

This study aimed to evaluate the antiplaque and antibacterial effects of a mouthwash containing *Lespedeza cuneata* (LC) extract through clinical periodontal disease (PD) indicators and changes in PD-causing bacteria.

**Methods:**

A total of 63 subjects participated in this double-blind clinical trial. Subjects were divided into two groups: 32 participants gargled with LC extract, and 31 used saline. Scaling was performed 1 week before the experiment to secure the homogeneity of the subjects’ oral conditions. After gargling with 15 ml of each solution for 1 min, participants spit out the solution to remove any residual mouthwash solution. Then, PD-related bacteria were measured via the O’Leary index, plaque index (PI), and gingival index (GI). The clinical data were collected three times: before gargling, immediately after gargling, and 5 d after gargling.

**Results:**

After 5 d, the O’Leary index, PI, and GI scores were significantly reduced in the LC extract gargle group (*p* < 0.05). PD-inducing Gram-positive and -negative bacteria were also reduced, confirming the LC extract’s effect on periodontal health improvement and disease prevention.

**Conclusion:**

Mouthwash containing LC extract, a new alternative natural substance that is safe and effective, may be used to treat PD because of its ability to inhibit and prevent PD.

## Background

Periodontal disease (PD) is an inflammatory disease affecting the supporting tissues of the teeth and characterized by the progressive destruction of periodontal ligaments, the formation of periodontal pockets of alveolar bone, and recession [1]. The prevalence of PD increases with age. According to the 2019 National Health Insurance Corporation’s Health Insurance Statistical Yearbook in South Korea, gingivitis and PD ranked as the most frequently occurring disease subcategories [2].

The proliferation of bacteria inhabiting the dental plaque and gingival sulcus is the main cause of PD, and they are classified into two complex groups according to the degree of pathogenicity and formation time: the red complex group with high-level risk, which includes *Porphyromonas gingivalis, Tannerella forsythia,* and *Treponema denticola*, and the orange complex group with medium-level risk, which includes *Prevotella intermedia* and *Parvimonas micra* [3, 4]. These bacteria directly affect periodontal tissue by producing a variety of toxins, which cause and exacerbate PD [5, 6]. Research on periodontal pathogens is being conducted using numerous approaches [7]. Furthermore, their effect on cardiovascular disease (CVD) risk has been demonstrated through the expression of gingival crevicular fluid (GCF) miRNA with periodontitis [8]. The physical method of brushing is mainly used for the removal of these PD-causing bacteria; however, if the environment or condition prevents effective biofilm removal, mouthwash can be used as an alternative [9]. Since chemical compounds in mouthwash often cause problems, research on natural extracts as ingredients with fewer side effects has been conducted [10, 11].

*Lespedeza cuneata* (LC) is a perennial belonging to the Leguminosae family and is widely distributed in East Asia. It has been studied as a green plant used for covering and improving wasteland and incised slopes [12]. It is also used in herbal remedies due to its efficacy in protecting the liver and kidneys, strengthening pulmonary function, and improving blood circulation [13, 14]. Furthermore, LC is used to cure asthma, boils, and breast cancer [15] and is effective at treating diabetes and male diseases, such as lack of stamina and sexual dysfunction [16]. β-sitosterol, quercetin, kaempferol, pinitol, avicularin, juglanin, and trifolin have been reported to be the active ingredients of LC [17], and various flavonoids have been found in the roots and leaves of LC [18]. Additionally, minerals, amino acids, and vitamins have been discovered in LC [13]. Ding et al. [19] analyzed the minerals and amino acids contained in LC and confirmed its high content of vitamin E, which has antiaging effects. Moreover, antibacterial and antioxidant effects [14, 20], skin photoaging inhibitory effects [21], skin whitening effects [22], skin photoaging improvement effects due to UV exposure [21, 23], wound healing effects [23, 24], blood sugar lowering effects, and cytoprotective effects against glucose toxicity [25] of LC have been reported.

According to Nam’s study [26], LC G. Don has a possible oral application of 10 mg/ml. When cytotoxicity was confirmed following application to oral epithelial cells in human keratinocytes (HaCaT), it demonstrated a safe and effective natural antifungal effect on *Candida albicans,* which causes oral candidiasis, the representative oral mucosal disease. Previous studies have reported several antibacterial effects of LC [13–26]. Thus, its effect on PD-causing bacteria can be hypothesized, but it is necessary to compare the effects of gargling with LC extract and saline on PD. Therefore, this study was conducted to evaluate the efficacy of LC extract as a mouthwash ingredient for inhibiting and preventing the progression of PD to maintain sound periodontal health by analyzing PD-related bacterial changes and clinical indicators.

## Materials and methods

### Ethics approval and consent to participate

The study was approved by the Institutional Review Board of Kangwon National University (KWNUIRB-2020-07-007-002, Chuncheon, South Korea) and registered as a clinical trial on the WHO International Clinical Trial Registry Platform (ICTRP) (registration date: 13/06/2022, registration number: KCT0007379; https://cris.nih.go.kr/cris/search/detailSearch.do/22017). Informed consent was obtained from all participants included in the study. All methods were carried out in accordance with the relevant guidelines and regulations. Additionally, this study was conducted in accordance with the International Council for Harmonization of Technical Requirements for Pharmaceuticals for Human Use (ICH) guidelines.

### Study design and protocol

This study was a randomized, double-blind, controlled clinical trial study. Among the patients who visited M dental clinic in Busan from October 2020 to June 2021, only those who gave consent after receiving an explanation regarding the study purpose from a dental hygienist with a minimum of 10 years of experience participated in this study. The participants were selected through an oral examination by a dentist according to the selection criteria. Subjects aged 19 years or older with more than 16 remaining teeth and severe dental diseases such as periodontitis or dental caries (one or more dental caries) were included. However, individuals with severe dental symptoms, such as dry mouth; those being treated for a systemic disease that may cause bad breath, such as liver disease, kidney disease, Sjogren’s syndrome, or rheumatism; smokers; those diagnosed with sinusitis and/or rhinitis; those taking antibiotics; those with tongue issues, including tongue cancer or glossitis; and those who had scaling within two months of the study period were excluded. Consequently, the number of participants in this study was 63.

### Study participants

**Fig. 1:**
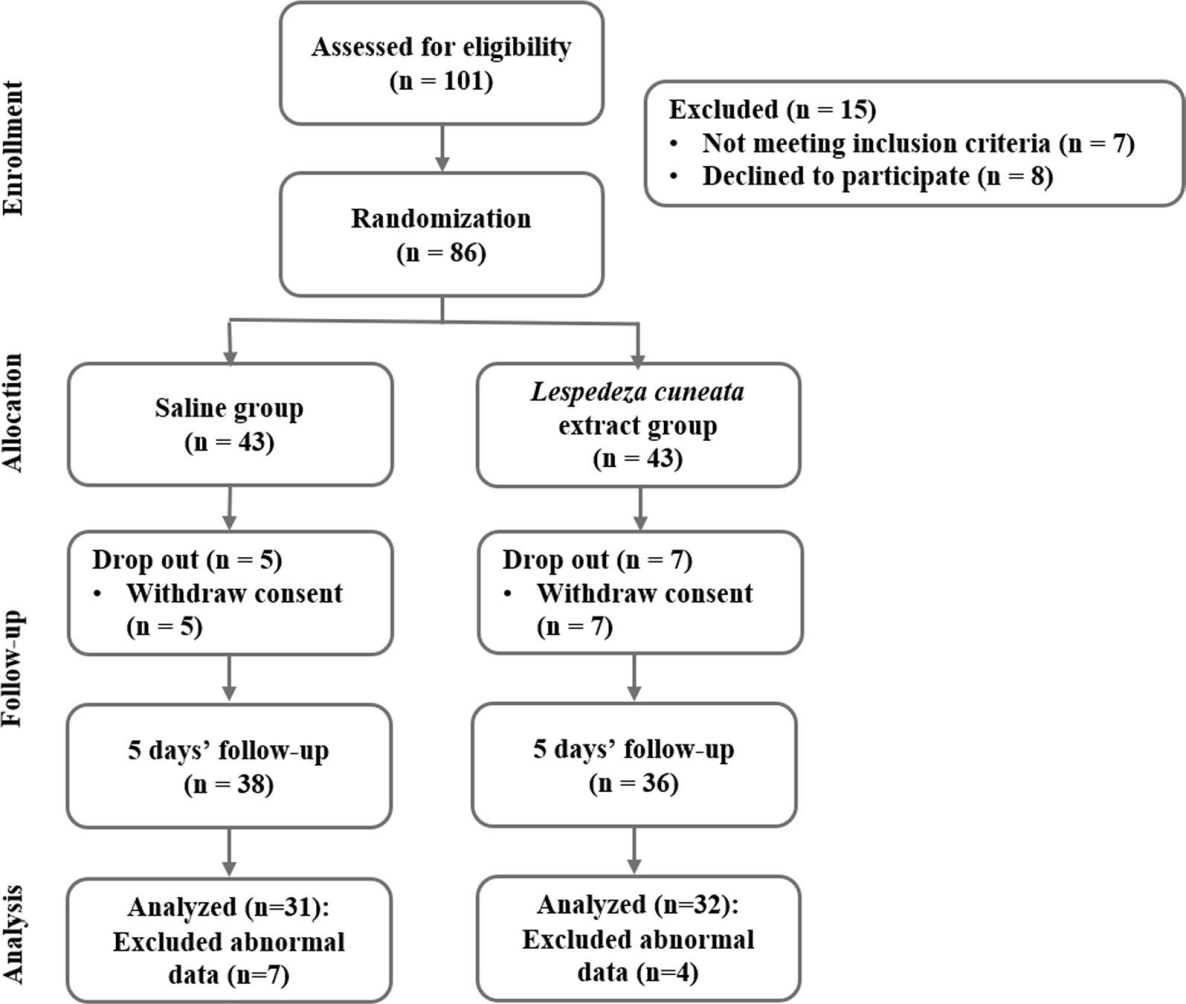
Flowchart of the study

The sample size was calculated using G*Power version 3.1 software. The number of participants needed for the independent t-test with a significance level of 0.05 in the bilateral test, a power of 0.8, and an effect size of 0.7 was 68. The planned sample size was 96, considering a dropout rate of 40%, and 100 participants were actually included in this study. The dropout rate was set fairly high because the subjects were college students or working adults. Out of a total of 101 subjects, 86 were selected, excluding 15 who declined to participate or did not meet the criteria. Subjects were then randomly grouped into two groups: a saline gargle group as the control group and an LC gargle group as the test group, with 43 people in each group. Consequently, 63 people were selected as final analysis subjects, excluding 12 subjects who did not complete the 5-d course and 11 subjects with insufficient data for analysis (Fig. 1).

### LC extract

The LC was purchased from Food Synergy Co., Ltd. (Seoul, South Korea). After 70% ethanol was added to the crushed LC, the extract was obtained after sitting at 60 °C for 12 h. The LC extract was filtered using qualitative filter paper, and the extract was concentrated using a rotary vacuum evaporator (N-1300E.V.S. EYELA Co., Tokyo, Japan). The LC was lyophilized using a freeze dryer at − 80 °C (Ilshin Lab Co., South Korea). The sample was prepared as a powder and stored at − 20 °C after dilution.

### Clinical examination

To ensure the homogeneity of the participants’ oral conditions, they received an examination by a dentist and light scaling from two dental hygienists at M dental clinic, Busan, 1 week prior to the study. One week after participants received scaling, the O’Leary index, plaque index (PI), gingival index (GI), and microbiological analysis results were evaluated as periodontal-related indicators via clinical examination. Furthermore, the experimental and control groups gargled for 1 min with 15 mL of mouthwash and saline, respectively, and clinical data were obtained immediately afterward. All participants were provided with the same toothbrush and toothpaste and with directions regarding the length and method of brushing required during the study. Participants were given mouthwash with a label that did not inform them which group they belonged to and were instructed to gargle with the mouthwash after brushing their teeth before bed. For a total of 5 d, the experimental group used 15 mL of a 10-mg/mL LC extract for 1 min, whose safety has been proven by Nam’s study [26], and the control group used 15 mL of saline. No food or drink, including water, was allowed after gargling. The O’Leary index, PI, GI, and microbiological analysis results were obtained by two dental hygienists who were educated under a dentist’s guidance for three periods: before treatment, immediately after treatment, and after 5 d.

#### O’Leary index

O’Leary, Drake, and Naylor’s dental plaque test (O’Leary index) was carried out [27]. We discolored all teeth in the oral cavity with a dental surface discoloration agent and calculated the level of adherence (%) using the plaque control score (O’Leary index), with a score of 1 if the plaque adhered to four tooth surfaces (mesial, distal, facial, and lingual) and 0 otherwise. The lowest score for one tooth is 0, and the highest score is 4, which is measured for all teeth except wisdom teeth.

#### PI

Using Loe and Silness’ PI technique [28], the maxillary right first molar (#16), maxillary left central incisor (#21), maxillary left first premolar (#24), mandibular left first molar (#36), mandibular right central incisor (#41), and mandibular right first premolar (#44) were selected as representative teeth and assessed. The tooth surface was divided into two parts by the gingival margin to measure plaque accumulation and thickness with a red colorant. The evaluation criteria were 0 for no plaque, 1 for plaque that was thinly attached to the gingival margin and apparent after light scraping with a probe or applying a tooth colorant, 2 for moderate plaque that could be visually recognized along the gingival margin, and 3 for thick plaque accumulation in the gingival pockets as well as the gingival margin and tooth surface. The PI score reflected the average amount of plaque per tooth surface measured, and the PI score for each tooth was calculated using the average value.

#### GI

Gingival health status was assessed using the GI technique [29]. The maxillary right first molar (#16), maxillary left central incisor (#21), maxillary left first premolar (#24), mandibular left first molar (#36), mandibular right central incisor (#41), and mandibular right first premolar (#44) were selected as representative teeth and assessed. The mesial, distal, buccal, and lingual areas of the tooth were measured. Each site was assigned a score from 0–3: 0 indicated normal gingiva; 1 indicated gingivitis with a slight color change and slight swelling but without bleeding caused by mild irritation; 2 reflected gingivitis with redness, swelling, and bleeding caused by mild irritation; and 3 indicated advanced inflammation with marked redness and swelling and the possibility of ulceration and natural bleeding. The total mean GI score for individuals was calculated by adding the values for each tooth.

#### Microbiological analysis

To obtain samples of subgingival microbiota from the periodontal pocket, sterilized #15 paper points were inserted in the gingival sulcus of two maxillary teeth (#16 and #21) and two mandibular teeth (#36 and #41) of subjects at a pocket depth (PD) of less than 4 mm for 10 s and then placed in a sterilized 1.5 ml tube. They were frozen at − 20 °C until just before analysis. DNA was extracted from the collected #15 paper points using the AccuPrep Universal RNA Extraction Kit (Bioneer, Daejeon, Korea). The extraction was performed according to the manufacturer’s instructions. OligoMix (YD Global Life Science Co., Ltd., Seongnam, Korea) and three oligonucleotides (forward primer, reverse primer, and probe) (Table 1) that react specifically to each bacterium were used [30]. Ten bacteria associated with PD were analyzed: *P. micra, Staphylococcus aureus (S. aureus), Eubacterium nodatum (E. nodatum), P. gingivalis, T. denticola, Fusobacterium nucleatum (F. nucleatum), P. intermedia, Prevotella nigrescens (P. nigrescens), Eikenella corrodens (E. corrodens), and Campylobacter rectus (C. rectus)*. For the preparation of the polymerase chain reaction (PCR) reaction sample, 9 μL of OligoMix, 10 μL of 2 × probe quantitative PCR (qPCR) mix (Takara Bio Inc., Shiga, Japan), and 1 μL of template DNA were combined. A 96-well plate containing the PCR reaction sample was placed in the CFX96 Touch Real-Time PCR Detection System (Bio-Rad, Hercules, USA) to amplify the DNA. The cycle conditions of PCR were as follows: initial activation for 30 s at 95 °C, denaturation for 10 s at 95 °C, and annealing for 30 s at 62 °C, with 40 repeated cycles. The cycle threshold (Ct) parameter was calculated using the Bio-Rad CFX Manager software, and the number of copies was calculated by plotting the Ct value in the standard curve for each bacterium.

**Table 1:** Primers and probes used in the real-time PCR assays

Bacteria	Target genes	Primers/probe sets	Amplicon size (bp)
*Parvimonas micra*	16S ribosomal RNA gene	5′-GAGGAATACCGGTGGCGAAG-3’ 5′-GGCACCGAGATTTGACTCCC-3’ 5′-FAM-GGTACGAAAGCGTGGGGAGCA-BHQ1-3’	148
*Staphylococcus aureus*	Clumping factor A (clfA) gene	5′-GCGCAAGTAACGAAAGCAAAA-3’ 5′-GATTTTGCGCCACACTCGTT-3’ 5′-FAM-TGCTGCACCTAAAACAGACGACACA-BHQ1-3’	132
*Eubacterium nodatum*	Hypothetical protein	5′-TGCTTGCCGGTGACTTAGGA-3’ 5′-AAACCGGGCTCAACAACCAT-3’ 5′-Texas Red-TTGAGGAGCCGGTGACTTTGG-BHQ2-3’	130
*Porphyromonas gingivalis*	Hemagglutinin (phg) gene	5′-ACACGGTGTATCGTGACGGC-3’ 5′-GCCGGCTGCGTACTTAACCT-3’ 5′-HEX-CGACCTACCGCGATGCAGGA-BHQ1-3’	119
*Treponema denticola*	Oligopeptidase B (opdB) gene	5′-AGAAAGGCTTTGGGCGACAG-3’ 5′-GCTGGAGCCGTAGCTTCCAT-3’ 5′-Cy5-CGGGTCCTCACCCGCTCTTC-BHQ2-3’	127
*Fusobacterium nucleatum*	16S ribosomal RNA gene	5′-GGCTGTCGTCAGCTCGTGTC-3’ 5′-CTCATCGCAGGCAGTATCGC-3’ 5′-FAM-AACGAGCGCAACCCCTTTCG-BHQ1-3’	114
*Prevotella intermedia*	hemagglutinin (phg) gene	5′-CACACGCTGGCGAAACCTAC-3’ 5′-CACGTGGCGTTGCTTCTTTC-3’ 5′-HEX-CCGAAGATGCGCCGTTGAAC-BHQ1-3’	143
*Prevotella nigrescens*	Gyrase subunit B (gyrB) gene	5′-AGCAAGCTGTAGGCGAGGCT-3’ 5′-GCTGAACACTTTCGCGTGCT-3’ 5′-Texas Red-GCTCGTATTGCAGCCCGCAA-BHQ2-3’	132
*Eikenella corrodens*	Proline iminopeptidase (pip) gene	5′-GCCAACTGCTGCTGGAAGTG-3’ 5′-GCCGCTGATTTCGGAGAGTT-3’ 5′-HEX- ACAGCCATCGGCACAGGCAT-BHQ1–3’	110
*Campylobacter rectus*	GroEL gene	5′-AAATTTAAGCGGCGACGAGG-3’ 5′-TCCTTGCTCACGCTTACGGA-3’ 5′-HEX-GGCTTTGACGCGGGCGTAGT-BHQ1-3’	132

### Statistical analysis

All clinically derived results were analyzed using SPSS 24.0 for Windows (IBM Corp., Armonk, NY, USA) at a significance level of 5%. The frequency of the participants’ demographic characteristics was analyzed, and the PD-related oral bacteria and clinical indicators of the saline mouthwash group and LC extract mouthwash group were compared via an independent t-test. The changes over time in each mouth, at baseline, immediately post-treatment (IPT), and 5 d post-treatment (FPT), were statistically analyzed using one-way ANOVA and Tukey’s post-hoc test.

## Results

### Population characteristics

The general characteristics of the participants are presented in Table 2. As for the gender distribution, the control group consisted of 25 women and 6 men, while the experimental group consisted of 26 women and 6 men, showing no significant difference (*p* > 0.05). The mean age of the subjects was 27.94 ± 8.40 years in the control group and 27.75 ± 8.33 years in the study group, showing no significant difference between the two groups (*p* > 0.05). In addition, no significant differences were observed between the groups in terms of systemic disease or marital status (*p* > 0.05).

**Table 2:** Characteristics of the participants in the saline and *Lespedeza cuneata* groups

Characteristics		N (%)		
		Saline	*Lespedeza cuneata* extract	*p-*value
*Gender	Male	6 (19.4)	6 (18.8)	0.951
	Female	25 (80.6)	26 (81.3)	
Age (mean ± SD)^￥^		27.94 ± 8.40	27.75 ± 8.33	0.930
*Systemic disease	No disease	28 (90.3)	29 (90.6)	0.967
	Have a disease	3 (9.7)	3 (9.4)	
*Marital status	Single	25 (80.6)	26 (81.3)	0.951
	Married	6 (19.4)	6 (18.8)	

^￥^*p*-values are determined by independent t-test

*p-values are determined by Chi-square test (*p* < 0.05). Values are means ± standard deviations; significant (bold)

### Measured clinical outcomes

The measurement results of PD-related clinical indicators between the saline mouthwash group and the LC extract mouthwash group are shown in Table 3. There was no difference (*p* > 0.05) between the two groups at baseline in terms of the O’Leary index, PI, or GI; however, significant differences were observed (*p* < 0.05) at IPT and FPT. The clinical efficacy of the LC extract was confirmed, as the group that used the mouthwash containing LC extract showed a lower level of PD-related indicators than the group that used the saline mouthwash. The saline group did not show any significant difference in measurement index t (*p* > 0.05) between the baseline, IPT, and FPT. In contrast, the LC extract group showed significantly lower levels of clinical indicators over time in the O’Leary index, PI, and GI (*p* < 0.05) (Table 3).

**Table 3:** Clinical outcomes observed between the groups

Variables	Group	Mean ± SD			
		Baseline	IPT	FPT	^*^p-value
O’Leary index	Saline	59.00 ± 6.30^a^	54.63 ± 7.20^a^	52.47 ± 6.41^a^	0.239
	*Lespedeza cuneata* extract	60.50 ± 7.21^a^	37.30 ± 4.43^b^	19.78 ± 5.31^c^	**0.000**
	^￥^*p-*value	0.673	**0.000**	**0.000**	
Plaque index (PI)	Saline	2.43 ± 0.25^a^	2.27 ± 0.34^a^	2.16 ± 0.35^a^	0.294
	*Lespedeza cuneata* extract	2.42 ± 0.20^a^	1.62 ± 0.1^b^	0.84 ± 0.24^c^	**0.000**
	^￥^*p-*value	0.912	**0.000**	**0.000**	
Gingival index (GI)	Saline	1.60 ± 0.33^a^	1.56 ± 0.32^a^	1.43 ± 0.26^a^	0.553
	*Lespedeza cuneata* extract	1.63 ± 0.26^a^	0.87 ± 0.20^b^	0.51 ± 0.14^c^	**0.000**
	^￥^*p-*value	0.846	**0.000**	**0.000**	

IPT; immediately post-treatment, FPT; 5 d post-treatment

^￥^*p*-values are determined by independent t-test

*p-values are determined by one-way ANOVA and Tukey’s post-hoc test (*p* < 0.05). Values are means ± standard deviations. Significant (bold); different letters (a, b, and c) indicate the statistically significant parameters

### Gram-positive bacteria in subgingival plaque

Table 4 shows Gram-positive bacteria in subgingival plaque. Three types of bacteria were observed: *P. micra*, *S. aureus*, and *E. nodatum.* In the case of *P. micra*, there was no significant difference (*p* > 0.05) in the maxilla, but a significant difference (*p* < 0.05) in the maxilla between the saline and LC extract groups at FPT was observed. *S. aureus* showed significant differences in both the maxilla and mandible at IPT and FPT, indicating a marked difference between the two groups (*p* < 0.05) (Table 4). Compared to the baseline, *P. micra, S. aureus*, and *E. nodatum* did not show any difference over time in the saline group; however, in the LC extract group, significant differences were observed in both the maxilla and mandible (*p* < 0.05) (Table 4).

**Table 4:** Gram-positive bacteria measurements in subgingival plaque

Variables	Group	Mean ± SD			
		Baseline	IPT	FPT	*p-value
*Parvimonas micra*					
Maxilla	Saline	6571.47 ± 14189.29^a^	428.00 ± 427.13^a^	372.00 ± 291.72^a^	0.310
	*Lespedeza cuneata* extract	5121.12 ± 2345.83^a^	288.13 ± 217.30^b^	283.63 ± 233.29^b^	**0.000**
	^￥^*p-*value	0.837	0.514	0.568	
Mandible	Saline	4872.63 ± 4510.54^a^	4500.46 ± 4397.13^a^	3842.83 ± 2866.92^a^	0.912
	*Lespedeza cuneata* extract	10785.19 ± 4745.23^a^	3942.70 ± 4161.90^b^	221.25 ± 224.66^b^	**0.000**
	^￥^*p-*value	0.068	0.869	**0.006**	
*Staphylococcus aureus*					
Maxilla	Saline	1285.59 ± 565.02^a^	525664.93 ± 269454.10^a^	333916.70 ± 173500.74^a^	0.238
	*Lespedeza cuneata* extract	441074.82 ± 227322.03^a^	104063.38 ± 60096.53^b^	96797.42 ± 53335.40^b^	**0.000**
	^￥^*p-*value	0.370	**0.000**	**0.004**	
Mandible	Saline	412635.35 ± 269375.13^a^	351940.78 ± 258647.71^a^	369800.30 ± 105396.48^a^	0.882
	*Lespedeza cuneata* extract	385448.22 ± 185448.29^a^	120766.60 ± 80220.88^b^	92382.15 ± 50117.54^b^	**0.000**
	^￥^*p-*value	0.834	**0.033**	**0.000**	
*Eubacterium nodatum*					
Maxilla	Saline	108.07 ± 260.15^a^	70.00 ± 50.04^a^	74.94 ± 44.78^a^	0.931
	*Lespedeza cuneata* extract	104.67 ± 76.78^a^	47.70 ± 37.93^a,b^	7.83 ± 6.64^b^	**0.002**
	^￥^*p-*value	0.975	0.409	**0.007**	
Mandible	Saline	2750.42 ± 3052.50^a^	2378.70 ± 1934.54^a^	1030.87 ± 941.59^a^	0.371
	*Lespedeza cuneata* extract	2237.59 ± 1658.03^a^	12.50 ± 8.29^b^	0.00 ± 0.00^b^	**0.000**
	^￥^*p-*value	0.731	**0.008**	**0.017**	

IPT; immediately post-treatment, FPT; 5 d post-treatment

^￥^*p*-values are determined by independent t-test

*p-values are determined by one-way ANOVA and Tukey’s post-hoc test (*p* < 0.05). Values are means ± standard deviations; significant (bold); different letters (a, b, and c) indicate the statistically significant parameters

### Gram-negative bacteria in subgingival plaque

As shown in Table 5, Gram-negative oral bacteria were observed in both groups. Seven types of bacteria—*P. gingivalis*, *T. denticola*, *F. nucleatum*, *P. intermedia*, *P. nigrescens*, *E. corrodens*, and *C. rectus*—were observed in both groups. In *P. gingivalis, P. intermedia,* and *C. rectus,* significant differences were found in both the mandible and maxilla at IPT and FPT in both mouthwash groups (*p* < 0.05). *T. denticola, P. nigrescens*, and *E. corrodens* showed differences between the two groups at IPT and FPT in the mandible only (*p* < 0.05), and in *F. nucleatum*, the difference between the two groups was observed at baseline in the maxilla and at FPT in the mandible (*p* < 0.05). When the changes over time from the baseline to FPT were measured, the saline group did not show any significant difference in any of the seven bacteria (*p* > 0.05). In comparison, the LC extract group showed significant differences at FPT in *P. gingivalis* and *P. intermedia*, in *P. nigrescens* (in the maxilla and mandible), in *F. nucleatum* (in the maxilla), and in *T. denticola* and *E. corrodens* (in the mandible) (*p* < 0.05) (Table 5).

**Table 5:** Gram-negative bacteria measurements in subgingival plaque

Variables	Group	Mean ± SD			
		Baseline	IPT	FPT	*p-value
*Porphyromonas gingivalis*					
Maxilla	Saline	1719.56 ± 284.35^a^	1726.71 ± 599.22^a^	1048.60 ± 420.30^a^	0.076
	*Lespedeza cuneata* extract	3021.33 ± 2393.37^a^	180.10 ± 274.77^b^	83.83 ± 102.91^b^	**0.000**
	^￥^*p-*value	0.174	**0.000**	**0.000**	
Mandible	Saline	1920.08 ± 1703.66^a^	2297.26 ± 483.09^a^	721.33 ± 204.71^a^	0.054
	*Lespedeza cuneata* extract	2029.07 ± 1885.78^a^	83.30 ± 126.58^b^	5.00 ± 6.15^b^	**0.001**
	^￥^*p-*value	0.914	**0.000**	**0.000**	
*Treponema denticola*					
Maxilla	Saline	668.41 ± 756.33^a^	537.96 ± 746.92^a^	426.93 ± 395.75^a^	0.784
	*Lespedeza cuneata* extract	519.12 ± 486.64^a^	264.04 ± 293.60^a^	128.41 ± 164.18^a^	0.100
	^￥^*p-*value	0.672	0.397	0.068	
Mandible	Saline	20147.48 ± 26483.85^a^	1178.00 ± 650.15^a^	846.63 ± 438.63^a^	0.073
	*Lespedeza cuneata* extract	12890.56 ± 13306.63^a^	377.60 ± 576.08^b^	101.60 ± 103.54^b^	**0.002**
	^￥^*p-*value	0.543	**0.024**	**0.002**	
*Fusobacterium nucleatum*					
Maxilla	Saline	763405.58 ± 354923.01^a^	545372.31 ± 279594.74^a^	574268.86 ± 218581.66^a^	0.336
	*Lespedeza cuneata* extract	1934684.25 ± 694857.37^a^	420170.67 ± 303094.92^b^	385497.04 ± 217478.71^b^	**0.000**
	^￥^*p-*value	**0.001**	0.439	0.122	
Mandible	Saline	1013721.57 ± 328581.04^a^	741980.57 ± 504432.09^a^	751504.38 ± 501037.69^a^	0.448
	*Lespedeza cuneata* extract	1219147.18 ± 1225868.33^a^	675757.00 ± 534901.44^a^	237264.43 ± 123110.06^a^	0.093
	^￥^*p-*value	0.935	0.907	**0.032**	
*Prevotella intermedia*					
Maxilla	Saline	2029.46 ± 1343.63^a^	1791.35 ± 735.74^a^	1344.32 ± 542.92^a^	0.464
	*Lespedeza cuneata* extract	2626.23 ± 1805.01^a^	8.00 ± 8.66^b^	0.00 ± 0.000^b^	**0.000**
	^￥^*p-*value	0.503	**0.000**	**0.000**	
Mandible	Saline	1166.32 ± 737.20^a^	1006.05 ± 336.18^a^	637.80 ± 181.07^a^	0.298
	*Lespedeza cuneata* extract	3940.83 ± 3644.60^a^	18.60 ± 28.38^b^	24.66 ± 24.86^b^	**0.000**
	^￥^*p-*value	0.052	**0.000**	**0.000**	
*Prevotella nigrescens*					
Maxilla	Saline	25892.85 ± 37364.94^a^	2217.38 ± 3075.79^a^	1667.09 ± 1797.62^a^	0.111
	*Lespedeza cuneata* extract	24803.89 ± 25047.38^a^	2112.11 ± 2487.72^b^	1598.11 ± 1459.22^b^	**0.005**
	^￥^*p-*value	0.956	0.948	0.941	
Mandible	Saline	28722.00 ± 12793.66^a^	24635.86 ± 12861.69^a^	3842.83 ± 2866.92^a^	0.304
	*Lespedeza cuneata* extract	25478.00 ± 30082.30^a^	2591.00 ± 2175.46^b^	24.66 ± 24.86^b^	**0.016**
	^￥^*p-*value	0.833	**0.001**	**0.002**	
*Eikenella corrodens*					
Maxilla	Saline	1285.59 ± 565.02^a^	969.14 ± 885.84^a^	916.05 ± 740.13^a^	0.731
	*Lespedeza cuneata* extract	1560.96 ± 1112.96^a^	1262.10 ± 969.28^a^	588.24 ± 482.03^a^	0.158
	^￥^*p-*value	0.613	0.579	0.375	
Mandible	Saline	389.85 ± 410.07^a^	292.48 ± 232.89^a^	62.84 ± 50.27^a^	0.179
	*Lespedeza cuneata* extract	201.37 ± 196.13^a^	0.00 ± 0.00^a^	0.00 ± 0.00^a^	**0.011**
	^￥^*p-*value	0.296	**0.009**	**0.014**	
*Campylobacter rectus*					
Maxilla	Saline	2527.86 ± 2798.43^a^	2906.74 ± 3130.46^a^	2218.55 ± 1478.12^a^	0.906
	*Lespedeza cuneata* extract	862.79 ± 584.37^a^	457.10 ± 497.13^a,b^	0.00 ± 0.00^b^	**0.002**
	^￥^*p-*value	0.127	**0.044**	**0.003**	
Mandible	Saline	5023.67 ± 31813.50^a^	35047.45 ± 31844.78^a^	13547.45 ± 11476.04^a^	0.084
	*Lespedeza cuneata* extract	58580.82 ± 33725.85^a^	0.00 ± 0.00^b^	0.00 ± 0.00^b^	**0.000**
	^￥^*p-*value	0.639	**0.024**	**0.016**	

IPT; immediately post-treatment; FPT; 5 d post-treatment

^￥^*p*-values are determined by independent t-test

*p-values are determined by one-way ANOVA and Tukey’s post-hoc test (*p* < 0.05). Values are means ± standard deviations; significant (bold); different letters (a, b, and c) indicate the statistically significant parameters

## Discussion

PD is caused when anaerobic bacteria proliferate on the surface of subgingival teeth and produce toxins harmful to periodontal tissues. Brushing is used to control the pathogen factor and manage dental plaque [31]. However, it is difficult to completely remove dental plaque with only physical brushing, so mouthwash containing antibacterial ingredients has been used in recent years [10, 11]. Therefore, this study investigated the anti-periodontitis effect of the natural ingredient LC extract through a randomized, double-blind, controlled clinical trial. The results of this study revealed a constant decrease in the O’Leary index, PI, and GI scores with LC extract-containing mouthwash compared to saline mouthwash. Apart from oral disease prevention, most people use mouthwash to freshen their breath or to combat bad breath. However, commercially available mouthwash with chemical ingredients may cause side effects such as drug resistance on long-term use, so it is necessary to create a mouthwash that is effective and safe for continuous use. After participants used the LC extract mouthwash for 5 d, the clinical indicators of PD improved, and the long-term usability of the mouthwash was confirmed. Furthermore, all three Gram-positive anaerobic bacteria showed a significant decrease after the use of the LC extract mouthwash compared to the saline mouthwash. Among all bacteria, *E. nodatum* is widely found in oral cavities with PD and is found to be strongly related to PD [32]. In this study, *E. nodatum* was not detected in the mandible at FPT with the LC extract mouthwash. Therefore, LC extract mouthwash is effective in decreasing Gram-positive anaerobic bacteria that are found deep inside the periodontal tissue and can serve as an excellent oral hygiene supplement for advanced PD.

In the case of Gram-negative bacteria, the red complex (*P. gingivalis* and *T. denticola)* was steadily reduced, and the orange complex (*P. intermedia)* was not detected in the maxilla at FPT with the LC extract mouthwash. Furthermore, *F. nucleatum,* which is found in adults with periodontitis and exhibits a synergistic effect with other pathogens in the periodontal pocket [33], decreased significantly at FPT with the LC extract mouthwash. Moreover, *P. nigrescens,* an oral bacterium that causes peri-implantitis, steadily reduced after the LC extract mouthwash use, which confirms its efficacy in PD due to implants. *P. nigrescens* causes not only peri-implantitis but also pulp and apical disease, periodontitis, and periodontal abscess and can have systemic effects, resulting in diabetes, myocardial infarction, premature birth, respiratory infection, urinary infection, brain abscess, osteomyelitis, rheumatoid arthritis, and other health problems [34]. PD bacteria related to these systemic diseases include *E. corrodens* and *C. rectus* [35]. *E. corrodens* causes arteriosclerosis, endocarditis, meningitis, respiratory infection, osteomyelitis, and other diseases and destroys periodontal tissues and hinders bone resorption. At IPT with the LC extract mouthwash, *E. corrodens* was not detected. Similarly, when compared to the baseline, there was no significant difference in *C. rectus*, as it was not detected in the oral cavity at FPT with the LC extract mouthwash. *C. rectus* causes fever, headache, muscle pain, diabetes, spontaneous abortion, and systemic food poisoning and is related to acute periodontitis in oral disease; thus, reducing the harmful effect of this bacterium is essential.

These results are similar to those of a study confirming the dental caries effect in an LC gargle compared to a saline gargle [36]. However, there are limitations on the comparison with this study. Therefore, studies to confirm various periodontal clinical indicators using gargles containing LC extracts are required. Furthermore, various types of research, such as those involving toothpastes, ointments, and tablets containing LC extract, along with extensive analysis of periodontal clinical indicators, are necessary. Based on the results of this study, LC extract showed potential as a natural mouthwash ingredient that can prevent and treat PD. As for the limitations of this study, the effect of LC extract on bacteria must be confirmed through a clinical study on severe PD patients. In addition, it is necessary to confirm the safety of long-term LC extract mouthwash use and further study its effect on various PD-causing bacteria. This clinical study is significant, as it verified the potential effect of the LC extract mouthwash despite a short-term application of 5 d. Therefore, LC extract is confirmed to have potential as a natural ingredient in mouthwash that improves the oral environment of PD patients and can be used in the prevention and treatment of PD in the clinical field.

## Conclusion

This study shows that LC extract-containing mouthwash improves the periodontal clinical indicators and reduces the bacteria involved in PD. Therefore, the results obtained can be used as basic data in the development of an antibacterial LC extract mouthwash for the treatment and prevention of PD, thereby contributing to the promotion of oral health.

## Data Availability

The datasets generated and/or analyzed during the current study are not publicly available for reasons of personal and organizational integrity but are available from the corresponding author on reasonable request.

## Abbreviations

PI: Plaque index
GI: Gingival index
*P. micra*: *Parvimonas micra*
*S. aureus*: *Staphylococcus aureus*
*E. nodatum*: *Eubacterium nodatum*
*P. gingivalis*: *Porphyromonas gingivalis*
*T. forsythia*: *Tannerella forsythia*
*T.** denticola*: *Treponema denticola*
*F. nucleatum*: *Fusobacterium nucleatum*
*P. intermedia*: *Prevotella intermedia*
*P. nigrescens*: *Prevotella nigrescens*
*E. corrodens*: *Eikenella corrodens*
*C. rectus*: *Campylobacter rectus*
IPT: Immediately post-treatment
FPT: 5 D post-treatment
LC: *Lespedeza cuneata*
PD: Periodontal disease
